# Effects of the healthy lifestyle community program (cohort 1) on stress-eating and weight change after 8 weeks: a controlled study

**DOI:** 10.1038/s41598-022-27063-4

**Published:** 2023-03-01

**Authors:** Corinna Anand, Karin Hengst, Reinhold Gellner, Heike Englert

**Affiliations:** 1 Faculty of Medicine, University of Muenster (WWU), Muenster, Germany; 2 Department of Food, Nutrition, Facilities, University of Applied Sciences Muenster, Corrensstraße 25, 48149 Muenster, Germany

**Keywords:** Nutrition, Public health, Weight management, Patient education, Disease prevention, Lifestyle modification, Preventive medicine

## Abstract

Stress-eating (eating more or more unhealthily in order to accommodate to stress), contributes to the development and maintenance of obesity. The effect of comprehensive weight loss interventions on changes in stress-eating as well as the contributing role of stress-eating on weight reduction has not been examined. The impact of the 8-week intensive phase of the Healthy Lifestyle Community Programme (HLCP, cohort 1) on emotional, external and restrained eating, as expressions of stress-eating was evaluated in a non-randomized controlled trial. Intervention: 14 seminars (twice per week, including practical units), complemented by stress-regulation and cooking workshops and coaching sessions empowering participants to change their behaviour towards a healthy plant-based diet (ad libitum), stress regulation, regular exercise and to focus on social support. Participants were recruited from the general population. In the intervention group, 91 participants (IG; age: 56 ± 10, 77% female) and in the control group, 52 (CG; age: 62 ± 14, 57% female) were enrolled. At baseline, participants of the IG reported higher levels of stress (9.7 ± 5.4 points [P] vs. 7.6 ± 6.2; p < 0.011), and of emotional eating (27.9 ± 9.4 vs. 20.0 ± 7.1; p < 0.001) and external eating (29.1 ± 4.9 vs. 25.5 ± 5.6; p < 0.001) than participants of the CG. Within 8 weeks, in the IG, scores of emotional eating (− 3.5 ± 5.4 P) and external eating significantly decreased (= − 2.0 ± 3.8 P), while restrained eating increased (2.7 ± 5.0 P; p for all < 0.001). Weight change was negatively correlated with change of external eating (R^2^ = 0.045; CC = − 0.285; p = 0.014), indicating that a greater weight change was associated with a smaller change of external eating. This is the first study to prospectively investigate the role of stress-eating on the weight reduction effect of comprehensive lifestyle interventions. Our data confirm that overweight is associated with EE and external eating and suggest that the HLCP is capable to reduce both, weight and stress-eating.

**Trial registration:** German Clinical Trials Register (DRKS; reference: DRKS00018821; September 18th 2019; retrospectively registered).

## Introduction

Chronic stress and overweight are of major public health relevance and there is a growing body of evidence that suggests that these two phenomena are connected^1–4^.

On the one hand, being overweight is associated with higher psychological stress^5^. For instance, people with overweight are confronted with psychosocial stress through weight stigma^6^, including prejudice, discrimination, and negative attitudes^7^. This is even the case for those who self-identify as being overweight but have a normal body mass index (BMI)^8^.

On the other hand, psychological stress is associated with overweight^9,10^. Stress contributes to the development and maintenance of obesity via multiple pathways, including physiological, psychological and behavioural mechanisms^10,11^. For instance, stress triggers physiological changes in the hypothalamic–pituitary–adrenal axis (e. g. cortisol dysregulation^12^) and reward processing in the brain and may induce overeating, especially of foods high in fat, sugar and energy^10^. As chronic positive energy balance is required to gain excessive weight, it is of great interest to understand how stress may contribute to overeating, which eventually leads to a positive energy balance^13^. There are three stress-related eating styles that indicate why people tend to eat more or more unhealthily in order to deal with the effects of stress:

Firstly, the concept of *emotional eating*, which is rooted in the psychosomatic theory^14,15^, describes eating in response to emotional stressors instead of internal signals of hunger or satiety^13,16^. Emotional arousal and stress may increase food intake in people with emotional eating behaviour^13^.

Emotional eating tends to co-occur with a second stress-related eating style: *external eating*, i.e. eating in response to environmental food cues such as picture of a well-known restaurant or the smell of tasty food^13,16^, again regardless of internal signals of hunger or satiety. People with emotional eating behaviour tend to shift their attention away from negative internal states by narrowing it to the immediate (food) environment, resulting in external eating^16^.

As a third relevant stress-related eating style, (3) *restrained eating*, i.e. eating less than desired to regulate body weight^16,17^, is of special interest in the context of obesity research. It is usually treated as a desirable and necessary trait in order to overcome overweight^18^. Yet, dieting and restraint can cognitively suppress feelings of hunger and appetite and are discussed to be a trigger for overeating^19,20^. Under stressful conditions, when cognitive control is disrupted by a stressor, people with restrained eating behaviour tend to eat more than non-dieters^13^. Thus, stress may lead to obesity by hindering the cognitive processes required for self-regulation and regulation of food intake in order to induce weight loss^7,10^.

Complicating matters, it is especially unhealthy energy-dense, highly palatable foods, high in sugar and fat, which are often eaten in response to stress^21^. Current evidence indicates that comfort food is capable of decreasing stress arousal^21^ as well as psychological^22^ and physiological stress^23,24^, and excessive intake typically leads to overweight^25^. Moreover, individuals with an unhealthy diet, e.g. low intake of vitamins and minerals, might have an increased susceptibility to stress^26^.

While comprehensive lifestyle intervention programs, including our own^27,28^, are suitable to induce weight loss and meet the complexity of sustainable behaviour change^29,30^, little is known about the effect of such programmes on stress-related eating behaviour and its influence on the effectiveness of the interventions in terms of improving body weight. Some, however, suggest that interventions which target emotional eating in a combined approach are effective for adults with overweight and/ or obesity who report elevated emotional eating^31^ and for individuals with diabetes^32^. Hence, weight loss interventions may improve eating behaviour^33^.

To our knowledge, there are no studies that have prospectively examined the mediating role of stress-eating on weight change in behavioural interventions. Few however, have examined the role of stress eating on weight change and therefore indicate that there may be a mediating effect^34–38^. For instance, studies suggest that mindfulness-based interventions support weight loss especially in participants who report higher levels of stress-related eating^34^, and positively affect overall metabolic health, e.g. weight and fasting glucose^35–38^. Moreover, a 6-month acceptance-based behavioural intervention contributed to significant weight reduction in adults with overweight, who had higher baseline levels of emotional eating (as one expression of stress-eating) in an uncontrolled pilot study^39^. Here, greater decreases in emotional eating were associated with even greater weight loss.

### Research gap

As described, a reduction of stress-eating is a promising target in the endeavours to combat obesity. To date, however, no studies have prospectively investigated the role of stress-related eating behaviour on the weight reduction effect of comprehensive healthy lifestyle intervention programs using standardized and validated instruments and assessing key variables of eating behaviour. Moreover, there are no evidence-based interventions to adequately consider the complexity of the interactions of stress, stress-eating and overweight. Holistic lifestyle approaches seem suitable to consider these interactions. As stress and obesity are highly prevalent in society today, innovative interventions should be conducted on a community-scale to improve the combined challenge of stress-related eating and obesity.

## Methods

### Study aim and hypotheses

We examined the effects of the holistic Healthy Lifestyle Community Programme (HLCP, cohort 1) on changes in stress-related eating behaviour, represented by emotional, external and restrained eating^13^, through the adoption of a healthy lifestyle, characterized by (1) good stress management^10,40^, (2) a healthy diet^41,42^, (3) regular physical activity^43^ and (4) social support^44^.

We hypothesized that weight change in participants with a low level of perceived stress would be more pronounced than in those with a high level of perceived stress.

We further hypothesized, that levels of emotional and external eating behaviour would be reduced and levels of restrained eating behaviour would be increased in participants of the intervention group (IG) after 8 weeks compared to baseline and compared to participants of the control group (CG).

Moreover, we hypothesized that changes in stress-eating would be positively correlated with weight change. A primary report on the effect of the HLCP regarding weight reduction and the metabolic risk profile of non-communicable diseases (NCDs) in the long-term is reported elsewhere^27^.

### Study design

This report is based on a secondary analysis after 8 weeks, based on a non-randomized, controlled intervention trial with a duration of 24 months^27^. The intervention in this study consisted of the HLCP (cohort 1). The control group received no intervention. In the main analysis^27^, the long-term effects of the HLCP on weight and cardiometabolic risk markers were evaluated.

### Study population

The sample size was calculated for the primary outcome of weight reduction^27^ and eligible participants were included into the present secondary analyses, accordingly. We recruited participants from the general population, as the HLCP followed a real-world community-approach, which has been described before^27^. Hence, we included not only participants with overweight and obesity but also persons who with normal-weight.

Participants of the intervention group and control group were recruited in two separate small municipalities (‘intervention municipality’ and ‘control municipality’) to keep the participants of the control group unaware of the lifestyle recommendations given to the intervention group. The complex real-world approach of our study, required involvement of local stakeholders within the ‘intervention municipality’^45^ in the planning stage and before recruitment was initiated, which is why randomization was not feasible (as described previously^27,46^). Accordingly, participants from the ‘control municipality’ did not receive the intervention.

As with all lifestyle interventions, blinding of participants or instructors to group allocation was not possible (as described previously^46,47^). The study was registered in the German Clinical Trials Register (DRKS; reference: DRKS00018821; www.drks.de, retrospectively registered).

Participants ≥ 18 years who were capable of understanding the study content were included. The study was conducted in accordance with the Declaration of Helsinki and was approved by the ethics committee of the Westphalia-Lippe Medical Association and the Muenster University (Muenster, Germany; reference: 2017-105-f-S; approved 5 April 2017). All participants provided written informed consent.

### Participants’ flow diagram

**Figure 1:**
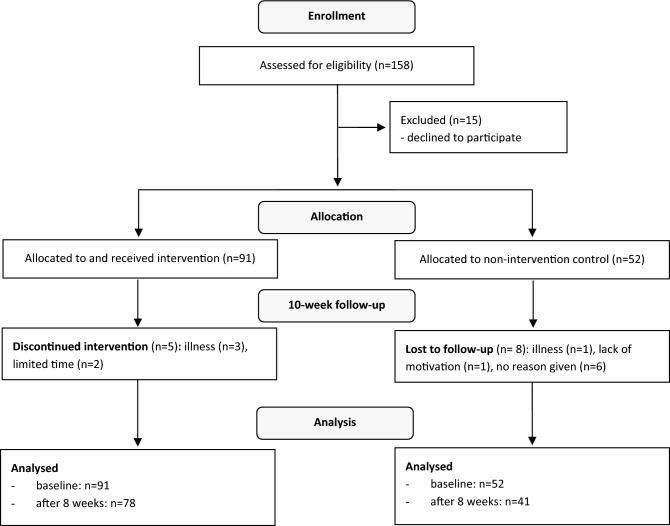
CONSORT structure participants’ flow diagram; participants categorized as “lost to follow-up” withdraw from the study with the given reason. In the IG, information is given on how many participants discontinued the intervention (e.g. dropped out) and why.

A participants’ flow diagram shows the study process from enrolment to analysis in Fig. 1. Before the onset of the study, 15 participants declined to participate. At baseline, 91 participants were included in intervention group and 52 in control group.

### Data assessment

#### Health check-ups

Baseline data were collected in April and October 2017 in intervention group and control group, respectively (see^27^), and equivalently in both groups after 8 weeks (i.e. the intensive phase of the HLCP in the intervention group). Unfavourably, it was not possible to recruit and start both study arms at the same time. This was due to the fact that funding was provided at relatively short notice, resulting in insufficient time and staff.

#### Anthropometric parameters

Body weight was determined by calibrated body scales, body height by self-report and BMI (kg/m^2^) was calculated accordingly. Waist circumference was measured according to the WHO protocol^48^.

#### Stress-related eating behaviour

Participants answered the German version of the Dutch Eating Behaviour Questionnaire (DEBQ)^13,17^, which assesses three dimensions of stress-related eating behaviour (33 items): (1) *emotional eating* (e.g., "Do you have the desire to eat when you are irritated?"), (2) *external eating* (e.g., "Do you eat more than usual when you see others eating?"), and (3) *restrained eating* (e.g., "Do you deliberately eat less in order to not become heavier?"). The instrument has been shown to have good reliability (emotional eating: Cronbach’s alpha [α] = 0.94, external eating: α = 0.89 restraint eating: α = 0.92) and construct validity, and to be suitable to reliably measure eating styles across age, gender, and BMI-status^13^. Responses were provided on a 5-point Likert scale ranging from 1 (seldom) to 5 (very often), resulting in maximal points of 65 for emotional, 50 for external, and 50 for restrained eating. Higher scores indicated a stronger expression of the respective eating behaviour.

#### Perceived stress level

In order to assess the psychosocial stress status, participants completed the German version of the Perceived Stress Scale-10 (PSS-10)^49^. Each item (e.g. “In the last month, how often have you felt that you were unable to control the important things in your life?”) was rated on a 5-point Likert scale ranging from 0 (never) to 4 (very often) resulting in maximal points of 40, with higher scores indicating higher levels of perceived stress. In order to compare high stress vs. low stress, participants who scored ≥ 14 on the PSS-10 at baseline were allocated to the high stress group^50^.

#### Further parameters

In the main analysis of the study^27^, additional parameters were assessed, including sociodemographic characteristics (e.g. age and sex), blood parameters (e.g. fasting glucose, insulin, lipids), vital parameters (e.g. blood pressure) as well as hormonal stress status (i.e. cortisol awakening response^47^) amongst others^51^.

#### Lifestyle intervention

The intervention was led by the study team and cooperating health care providers (e. g. local practitioners) and has been described in detail previously^27^. In short, the 8-week intensive phase of the comprehensive HLCP started and ended with an individual health check-up, including the above-mentioned parameter assessment, and a personal coaching session for every participant. Here, health check-up results, personal goals and options for lifestyle and eating behaviour change were discussed.

After the coaching sessions, the intensive phase continued with of 14 consecutive seminars (twice per week for 2 h each) with a strong emphasis on the potential of behaviour change, perception of internal signals (e.g. appetite, hunger, frustration, stress or eating with pleasure) and community support as well as improvement of self-efficacy and relapse prevention. The four main topics of lifestyle change included a healthy, predominantly plant-based diet, stress regulation, physical activity, and social health^27^. One of the 14 seminars was exclusively about stress as a health factor, including information about stress-eating. Exercises on stress regulation and mindful eating (e.g. ‘eating meditation’) were integrated into all seminar sessions. Moreover, participants were guided to reflect their own eating behaviour and to change towards healthy, more plant-based dietary patterns using vivid study material (e.g. recipe book, training manual, pleasure exercises). Crucially, participants were empowered to improve their practical cooking and food preparation skills (e.g. presentation of healthy food and live-preparation of healthy plant-based recipes within the seminars), Additionally, cooking workshops (goal: try out and learn new healthy recipes with like-minded people) and *stress-and-eating*-workshops (goal: reflect persona eating behaviour in the context of stress in an environment of trust ) were offered in smaller groups (~ 20 participants; ~ 2-h duration) to allow for more room for individual self-reflection and change strategies as well as to strengthen group support.

After the intensive phase of 8 weeks, a subsequent 22-months-alumni-phase with a less intensive intervention followed. Here, participants joined monthly meetings (2 h each) in which contents of the intensive phase were refreshed and group support was strengthened. Notably, results of this follow-up phase are not part of this sub-study.

#### Control group (CG)

Participants of the control group did not receive any intervention. For ethical reasons, they were informed about their health check-up results and were offered to participate in a subsequent HLCP after completion of the study.

### Statistics

Continuous variables are presented as mean ± standard deviation (SD), categorical variables as frequencies and valid percent. Normality was tested using the Shapiro–Wilk test and judged by histograms. All data were analysed in accordance with the predefined study plan. All available data were analysed. Missing data were not imputed.

To compare intervention group and control group, independent t-test was used for normally distributed continuous variables (e.g. changes of eating behaviour scores) and Mann–Whitney *U* test as its nonparametric alternative. For dichotomous variables, Fisher`s exact test was used. Within-group comparisons were performed by one sample *t*-test for normally distributed variables. Otherwise the nonparametric Wilcoxon signed-rank test was performed. All tests were two-sided. Relations between two continuous variables (e.g. weight change and change of emotional eating) were assessed with Spearman correlation coefficient (two-sided).

Subgroups were formed to separately analyse the weight reduction effects of the intervention in participants who were overweight and normal-weight and in participants with high and low stress levels.

Multiple linear regression modeling (MLR) was used to explore the effect of the intervention on the change of stress eating dimensions, adjusting for sex and the baseline value of the respective variable. Variables found to be associated with change of DEBQ and PSS-10 parameters were also added as covariates (in addition to the group variable) to the multiple regression model using a forward–backward selection approach. Regression models that were statistically relevant (p ≤ 0.05), with the highest corrected R^2^, and the lowest number of covariates were selected. In all models, residuals have been checked for normality.

p-values < 0.05 are considered significant, and are to be understood as exploratory^52^ because all presented data are secondary outcome parameters. All available cases were used.

Statistical analyses were performed using IBM SPSS Statistics for Windows, Version 27.0. Armonk, NY: IBM Corp.

### Ethics approval and consent to participate

The study was conducted in accordance with the Declaration of Helsinki and the study protocol was reviewed and approved by the ethics committee of the Westphalia-Lippe Medical Association and the Muenster University (Muenster, Germany; approval number 2017-105-f-S; approved April 5th 2017). All participants provided written informed consent.

## Results

### Baseline characteristics of the intervention and control group

Participants of the intervention group attended 9 out of 14 seminars (63%), on average. Baseline characteristics of intervention group and control group are shown in Table 1. The groups showed statistically relevant differences: participants of the intervention group were younger (p < 0.001), more often female (p = 0.024), had a higher BMI (p = 0.020) and were more often overweight (p = 0.015). They also reported higher levels of emotional (p < 0.001) and external eating behaviour (p < 0.001) as well as a higher average perceived level of stress (p = 0.011).

**Table 1:** Baseline characteristics by study group (n = 143).

	Intervention	Control	p-value
	n = 91	n = 52	
**Sociodemographics**			
Age, mean ± SD	56 ± 10	62 ± 14	** < 0.001**^a^
Female, n (%)	70 (77)	31 (57)	**0.024**^b^
**Anthropometrics**	n = 90	n = 51	
Weight (kg), mean ± SD	85 ± 19	80 ± 18	0.113^a^
BMI (kg/m^2^), mean ± SD	29 ± 5	27 ± 7	**0.020**^a^
Participants with overweight/obesity^c^, n (%)	67 (74)	28 (55)	**0.015**^b^
WC (cm), mean ± SD	98 ± 14	98 ± 17	0.770^a^
Participants with elevated WC^d^, n (%)	60 (67)	31 (61)	0.465^b^
**Psychosocial stress parameters**	n = 89	n = 49	
Stress-related eating scores^e^, mean ± SD	n = 88	n = 48	
Emotional eating behaviour	27.9 ± 9.4	20.0 ± 7.1	** < 0.001**
External eating behaviour	29.1 ± 4.9	25.5 ± 5.6	** < 0.001**
Restrained eating behaviour	28.7 ± 6.5	28.9 ± 7.6	0.547
Perceived stress score^f^, mean ± SD	9.7 ± 5.4	7.6 ± 6.2	**0.011**^a^
High stress level^g^, n (%)	23 (25)	11 (21)	0.684^2^

Significant values are in bold.

*BMI* Body Mass Index, *WC* Waist circumference, ^a^Mann–Whitney-*U* test, ^b^Fisher’s exact test, ^c^BMI ≥ 25 kg/m^2^, ^d^≥ 102 cm and ≥ 88 cm for men and women, respectively, ^e^Dutch Eating Behavior Questionnaire^13^.^f^Perceived Stress Scale (PSS)-10 score^49^, ^g^PSS-10 score ≥ 14.

### Association of stress, stress-eating and weight status (both groups combined)

Spearman correlation analyses revealed no significant correlation of the BMI and perceived stress level (p = 0.065; see Table 2). But highly stressed participants (PSS-10 ≥ 14 points) of both groups combined (n = 32) had a higher BMI (31 ± 8 kg/m^2^) than those with a low stress level (27 ± 6 kg/m^2^; p = 0.006).

**Table 2:** Association between Body Mass Index (BMI), perceived stress (PSS-10) and stress-related eating behaviour in the intervention and control group (combined).

	n =	BMI		perceived stress level	
		CC	p =	CC	p =
Perceived stress level	138	0.158	0.065	–	–
Stress-related eating					
Emotional eating	117	0.326	** < 0.001**	0.520	** < 0.001**
External eating	117	0.264	**0.002**	0.344	** < 0.001**
Restrained eating	135	0.013	0.885	− 0.005	0.952

Significant values are in bold.

*BMI* Body Mass Index (kg/m^2^), *CC* Pearson correlation coefficient, *p* p-value.

Due to sex-specific reference values, men and women were analysed separately regarding the connection of stress level and waist circumference ([WC] high-risk WC for men ≥ 102 cm and for women ≥ 88 cm, respectively). Women with a high level of perceived stress more often had a high-risk waist circumference (86% [n = 24]) than those with a low stress level (61% [n = 43]; p = 0.029). In men, we detected no statistically relevant connection (p = 0.122). However, only 4 men had a high-risk waist circumference and all of them reported a high stress level at baseline.

Accordingly, male and female participants with a high perceived stress level had a higher WC than those with a low stress level (men: 134 ± 20 vs. 104 ± 11; p = 0.008; women: 99 ± 13 vs. 93 ± 14; p = 0.049). In men, a higher waist circumference was associated with external eating (correlation coefficient [CC] = 0.346; p = 0.029), whereas in women higher waist circumference was associated with a higher emotional eating score (CC = 0.265; p = 0.010).

A higher perceived stress level was also associated with higher scores of emotional and external eating behaviour, but not of restrained eating behaviour (see Table 2).

Participants who were overweight (n = 91) scored higher than participants with normal weight (n = 45) in emotional eating behaviour (27 ± 10 points [P] vs. 21 ± 8 P; p = 0.002) and external eating behaviour (29 ± 5 P vs. 26 ± 5 P; p = 0.010). The positive association between the BMI, emotional and external eating behaviour was also confirmed by Spearman correlation, but higher restrained eating behaviour was not linked to lower BMI (Table 2).

### Changes of weight, stress level and stress-eating after 8 weeks

As described before^53^, in the intervention group, weight decreased significantly after 8 weeks (− 1.5 ± 1.9 kg) compared to baseline (p < 0.001), significantly more than in control group (− 0.3 ± 1.7 kg; p < 0.001), and more distinctly in participants with overweight (− 1.8 ± 2.0 kg)^53^. Average perceived stress levels decreased after 8 weeks in the intervention group and the control group, resulting in no differences between the groups (Table 3).

**Table 3:** Change of perceived stress and stress-related eating behaviour after 8 weeks in participants with normal weight and overweight of the intervention and control group.

	Intervention group			Control group			BGC
	Baseline	Change	p^#^ =	Baseline	Change	p# =	p^$^ =
Normal weight	n = 23	n = 20		n = 23	n = 20		
Perceived stress level	9 ± 4	− 3 ± 4	**0.002**	7 ± 5	− 2 ± 4	**0.024**	0.376
Emotional eating	23 ± 8	− 4 ± 5	**0.002**	20 ± 7	+ 2 ± 3	0.189	** < 0.001**
External eating	28 ± 5	− 2 ± 3	**0.027**	25 ± 5	− 1 ± 3	0.550	0.246
Restrained eating	28 ± 7	+ 2 ± 6	0.121	29 ± 9	+ 2 ± 5	0.282	0.661
Overweight	n = 66	n = 58		n = 26	n = 21		
Perceived stress level	10 ± 6	− 3 ± 5	** < 0.001**	8 ± 7	− 2 ± 4	**0.039**	0.841
Emotional eating	30 ± 9	− 3 ± 6	** < 0.001**	20 ± 8	− 1 ± 3	0.165	0.110
External eating	29 ± 5	− 2 ± 4	** < 0.001**	26 ± 6	− 1 ± 2	0.165	0.142
Restrained eating	29 ± 7	+ 3 ± 5	** < 0.001**	29 ± 6	0 ± 4	0.223	**0.025**

Significant values are in bold.

Normal weight (BMI < 25 kg/m^2^); Overweight (≥ 25 kg/m^2^); w = week; p^#^ = within group p-value; p^$^ = between group p-value; *BGC* between group comparison.

In the intervention group, but not in the control group (p > 0.158), participants with a low stress level and who were overweight (n = 45) lost more weight (− 2.0 ± 2.1 kg) than those with a low stress level who were of normal-weight (n = 18; − 0.9 ± 1.1 kg; p = 0.016). There was no difference in weight reduction within the intervention group between participants with high and low stress levels (p > 0.202). Notably, the change of weight was neither associated with a change of perceived stress in all participants (IG: correlation coefficient [CC] = − 0.034; p = 0.765; CG: CC = − 0.178; p = 0.307) nor in subgroups of participants with overweight (IG: CC = 0.044; p = 0.742; CG: CC = − 1.112; p = 0.657) or normal-weight (IG: − 0.044; p = 0.853; CG: CC = − 0.207; p = 0.425).

**Figure 2:**
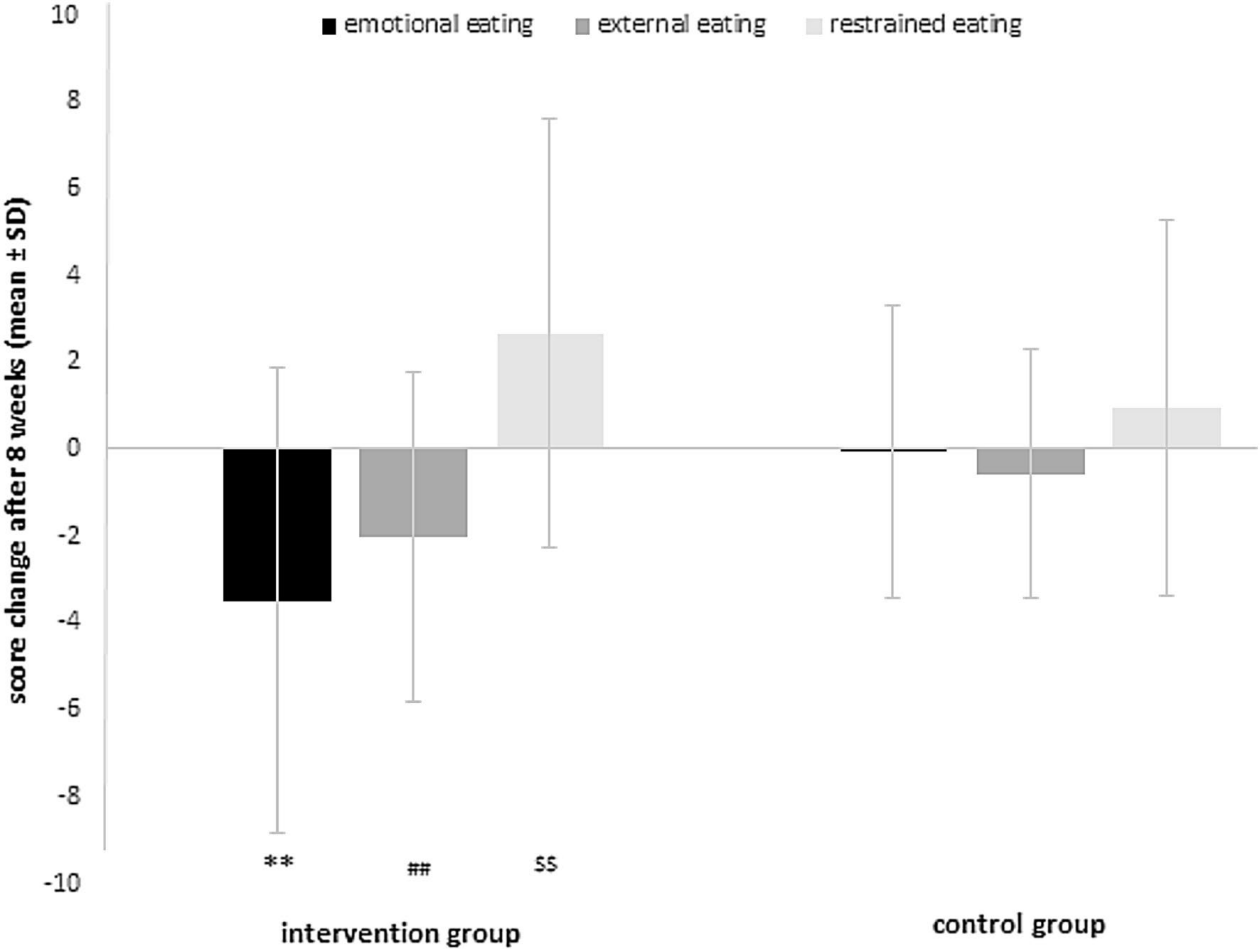
Change of stress-related eating behaviour in the intervention group (IG) and control group (CG); Changes of stress-eating scores: emotional eating: IG = − 3.5 ± 5.4 points (P); CG = − 0.05 ± 3.4 P; external eating: IG = − 2.0 ± 3.8 P; CG = − 0.6 ± 2.9 P; restrained eating: IG = 2.7 ± 5.0 P; CG = 1.0 ± 4.3 P. legend: **^,##,$$^p < 0.001. compared to baseline; **p < 0.001, ^##^p = 0.042, ^$$^p = 0.024 compared to CG.

We did, however, observe a significant change of all scores of stress-related eating behaviour, i.e. a decrease of emotional and external eating and an increase of restrained eating in the intervention group (for all p < 0.001), but not in the control group, resulting in relevant between-group differences (p < 0.05; Fig. 2). Restrained eating behaviour in the intervention group changed significantly only in participants with overweight, but not in participants who were normal-weight (see Table 3).

**Figure 3:**
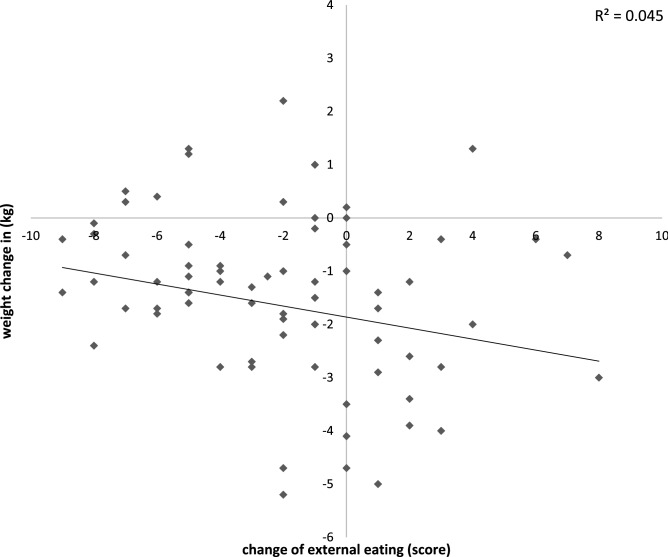
Correlation of the change of external eating and weight change in participants of the intervention group (n = 78) after 8 weeks; Spearman correlation coefficient (CC) = 0.285; p = 0.014.

As correlation analyses revealed, the weight reduction in the intervention group was not associated with a change of emotional (p = 0.537) or restrained eating (p = 0.335). However, weight change was negatively correlated with external eating (R^2^ = 0.045; CC = − 0.285; p = 0.014; see Fig. 3) and this was even more pronounced in the overweight subgroup (R^2^ = 0.089; CC = − 0.535; p < 0.001). These results indicate that a greater weight change was associated with a smaller change of external eating behaviour. In further analyses, we found participants with lower baseline values of external eating tended to have a smaller decrease or even increase of external eating after 8 weeks (R^2^ = 0.173; CC = − 0.442; p < 0.001). Against this background, we tested if participants with a higher weight reduction, i.e. less change of external eating, had lower baseline values of the external eating score, but this was not the case (R^2^ = 0.005; CC = 0.049; p = 0.673).

### Multiple linear regression modelling (MLR)

When adjusting for the baseline value of the respective variables, MLR revealed no significant impact of the HLCP on emotional (corrected [corr.] R^2^ = 0.274; ß = − 1.438; p = 0.121) or external eating behaviour (corr. R^2^ = 0.123; ß = − 0.603; p = 0.386), but indicated an effect on restrained eating behaviour (corr. R^2^ = 0.203; ß = 1.609; p = 0.049).

However, the changes of the three stress-related eating styles were not predictive for weight loss in the intervention group (emotional eating: p = 0.500; external eating: p = 0.071; restrained eating: p = 0.101). In the control group, weight gain was predicted by an increase of emotional eating (corr. R^2^ = 0.219; ß = − 0.252; p = 0.004).

The change of perceived stress did not qualify as a predictor for weight change (p > 0.754).

## Discussion

The Healthy Lifestyle Community Program (HLCP, cohort 1) resulted in a change of stress-related eating behaviour after 8 weeks, which was measured by emotional and external eating, which decreased, and restraint eating, which increased. This is in line with our hypotheses before the onset of the study. Although, multiple linear regression modelling did not confirm a significant impact of the HLCP on emotional and external eating behaviour, its impact on restrained eating was significant. Moreover, we found that a greater weight reduction was associated with a smaller change of external eating scores in the intervention group, especially in participants who were overweight.

In the control group, an increase of emotional eating was predictive for weight gain.

In our sample, a higher BMI was linked to higher levels of emotional and external eating behaviour. Scores of emotional and external eating decreased significantly in the intervention group compared to baseline and more than in the control group. As these two eating styles are related to overeating in stressful situations and to overweight^16,54^, the reduction is considered to support weight loss efforts^55^. Moreover, a reduction of external eating is desirable, because ubiquitously available energy-dense and highly palatable comfort foods challenge people with external eating behaviour^25^.

Here, a more intuitive and mindful eating behaviour may contribute to the reduction of stress-eating^56,57^. In accordance with our results, other interventions, combining mindfulness and cognitive behavioural approaches also resulted in reduced external^35,58–60^ and emotional eating^55,56^. As O’Reilly and colleagues^57^ summarized, interventions that reduced external eating typically included mindful eating components, which was also the case in the HLCP, i.e. exercises on these topics were incorporated in the HLCP seminars and workshops. However, in our multiple regression analyses, the group variable was not predictive for the change of stress-eating. A stronger emphasize on mindful eating might have led to clearer results here.

And yet, although we observed a reduction of emotional and external eating as well as weight^53^ in the intervention group, correlation and regression analyses did not reveal that the changes of eating behaviour favoured weight loss. To the contrary, we found a negative association of the changes of external eating and weight, indicating that those participants who lost more weight changed external eating to a smaller extend than those who lost less or gained weight. These results are not consistent with the literature in this field. Existing evidence clearly suggests, that an increase of external eating is associated rather with weight gain than with weight loss^17,35,57^. Compared to others, the change of external eating was relatively modest in our study. For instance, an eight-week healing meditation resulted in -7.9 points in women with overweight and obesity^61^. Assuming that participants with greater weight change may have started with low levels of external eating and therefore may have had smaller scope for change, was also not backed up by our analyses.

Moreover, the mediating effect of changes in stress-eating on weight change should be clarified in mediation models to give insights in the complex interaction of stress-related eating behaviour and weight reduction in the context of comprehensive lifestyle interventions.

Notably, the intervention group started with significantly higher levels of emotional and external eating behaviour than the control group. This might have influenced the results, as adjusting for baseline values did not identify the HLCP to be a significant predictor of stress-eating changes. Further research with more comparable groups may give valuable insights into the effect of the HLCP on emotional and external eating behaviour and their impact on weight change.

Importantly, the role of restrained eating behaviour as a dimension of stress-eating needs to be further explored. It is generally agreed, that cognitive control of food intake is necessary to successfully reduce weight, and results from the German Weight Control Registry underline, that weight-loss maintenance is associated with higher dietary restraint^62^. In line with this, our results indicate that the increase of restrained eating contributed to the weight loss effect of the HLCP. But, individuals with restrained eating behaviour are more likely to overeat when it comes to stressful events. For instance, a Malaysian study with nurses (n = 1022) documented that participants who were overweight tried to achieve an ideal weight by reducing their food intake, which, however, led to weight gain instead^20^. Additionally, dietary restraint may leave individuals vulnerable to emotional eating in stressful situations^19^. As the three discussed dimensions of stress-related eating behaviour may reinforce each other, the individual may end up stuck in a vicious cycle^62^. Tomiyama concluded, that stress-induced eating may be difficult to eliminate because highly palatable foods are easily accessible, and eating is pleasurable and may therefore serve as a tempting coping mechanism to escape stressful situations^10^. Hence, holistic approaches for lifestyle change, providing comprehensive behavioural measures to reduce weight and stress-related eating behaviour are a great challenge and of great importance.

Moreover, rigid restraint may lead to the above-mentioned vicious cycle, but flexible restraint was shown to not promote weight gain^63^ and to predict better long-term weight loss^64^. Hence, encouraging flexible restraint may be important in preventing and treating obesity^65^. However, we did not differentiate between rigid and flexible restraint in this study. In further research, measuring different types of restrained eating may give insight on whether the increase in restrictive eating behaviour was a healthy or unhealthy effect of the HLCP.

In accordance with other studies^66–70^, our analyses indicate that a high perceived stress level is associated with overweight, higher waist circumference and higher levels of emotional and external eating behaviour. Underlining this, we observed that higher levels of emotional and external eating were associated with higher BMI and were more often reported by participants who were overweight, as it was described before^71^. Yet, we did not find the change of perceived stress to be associated to greater reduction of weight parameters. Significant interactions between changes in chronic stress and group condition, may indicate that a greater decrease of chronic stress may be related to greater decreases in weight parameters, as Daubernmier et al. (2011) suggested for abdominal fat^35^. Yet, we also did not find, that the intervention reduced perceived stress levels noteworthily compared to the control group^51^, allowing no such conclusion.

Some limitations of the study have to be mentioned. First, recruiting from the general population with regard to participation in the Healthy Lifestyle Community Program (HLCP, cohort 1) may have contributed to the considerable higher baseline levels of emotional and external eating, BMI and perceived stress in the intervention group (IG). Despite efforts to enrol comparable participants in both groups, it seems possible that recruitment of participants in the intervention group was influenced by the fact that they had a greater need for interventions to improve weight, eating behaviour, and their general chronic disease risk profile. Second, the mostly female sample might limit the generalizability of these findings. Third, in statistical analysis, subgroups (i.e. normal and overweight as well as high and low stress level) were built retrospectively and were not considered in the calculation of the sample size. Future studies examining the effect of the HLCP on stress-related eating should aim for a replication in a larger sample.

## Conclusions

Our study is the first one to prospectively investigate the role of stress-related eating behaviour on the weight reduction effect of comprehensive healthy lifestyle intervention programs using standardized and validated instruments and assessing key variables of eating behaviour. Our data confirm the assumption that overweight is associated with higher levels of perceived stress as well as emotional and external eating and suggest that the Healthy Lifestyle Community Program (HLCP, cohort 1) may reduce the same, and increases dietary restraint. The impact of these changes on weight loss have to be further explored. Our findings underline the need to consider stress-associated eating behaviour in holistic weight loss interventions to account for the complex association of chronic stress, overweight, and obesity.

## Data Availability

The data are available from the corresponding author (CA) upon reasonable request.

## Abbreviations

BGC: Between group differences
BMI: Body mass index
CC: Correlation coefficient
CG: Control group
corr.: Corrected
DEBQ: Dutch Eating Behaviour Questionnaire
EE: Emotional eating behaviour
ExE: External eating behaviour
HLCP: Healthy Lifestyle Community Programme
IG: Intervention group
MLR: Multiple linear regression modelling
NCDs: Non-communicable diseases
NW: Normal weight
OW: Overweight
P: Points
PSL: Perceived stress level
PSS-10: Perceived stress scale-10
RE: Restrained eating behaviour
SD: Standard deviation
WC: Waist circumference
WHO: World Health Organisation
